# Viral community analysis in a marine oxygen minimum zone indicates increased potential for viral manipulation of microbial physiological state

**DOI:** 10.1038/s41396-021-01143-1

**Published:** 2021-11-06

**Authors:** Sophie K. Jurgensen, Simon Roux, Sarah M. Schwenck, Frank J. Stewart, Matthew B. Sullivan, Jennifer R. Brum

**Affiliations:** 1 Department of Oceanography and Coastal Sciences, Louisiana State University, Baton Rouge, LA 70803 USA; 2 DOE Joint Genome Institute, Lawrence Berkeley National Laboratory, Berkeley, CA 94720 USA; 3 Scripps Institution of Oceanography, University of California San Diego, La Jolla, CA 92093 USA; 4 Microbial and Environmental Genomics Group, J. Craig Venter Institute, La Jolla, CA 92037 USA; 5 Department of Microbiology & Cell Biology, Montana State University, Bozeman, MT 59717 USA; 6 School of Biological Sciences, Georgia Institute of Technology, Atlanta, GA 30332 USA; 7 Departments of Microbiology and Civil, Environmental, and Geodetic Engineering, The Ohio State University, Columbus, OH 43210 USA

**Keywords:** Microbial biooceanography, Microbial ecology

## Abstract

Microbial communities in oxygen minimum zones (OMZs) are known to have significant impacts on global biogeochemical cycles, but viral influence on microbial processes in these regions are much less studied. Here we provide baseline ecological patterns using microscopy and viral metagenomics from the Eastern Tropical North Pacific (ETNP) OMZ region that enhance our understanding of viruses in these climate-critical systems. While extracellular viral abundance decreased below the oxycline, viral diversity and lytic infection frequency remained high within the OMZ, demonstrating that viral influences on microbial communities were still substantial without the detectable presence of oxygen. Viral community composition was strongly related to oxygen concentration, with viral populations in low-oxygen portions of the water column being distinct from their surface layer counterparts. However, this divergence was not accompanied by the expected differences in viral-encoded auxiliary metabolic genes (AMGs) relating to nitrogen and sulfur metabolisms that are known to be performed by microbial communities in these low-oxygen and anoxic regions. Instead, several abundant AMGs were identified in the oxycline and OMZ that may modulate host responses to low-oxygen stress. We hypothesize that this is due to selection for viral-encoded genes that influence host survivability rather than modulating host metabolic reactions within the ETNP OMZ. Together, this study shows that viruses are not only diverse throughout the water column in the ETNP, including the OMZ, but their infection of microorganisms has the potential to alter host physiological state within these biogeochemically important regions of the ocean.

## Introduction

Marine viruses are now recognized to play key roles in marine ecosystems by lysing ~10–40% of bacteria per day, recycling organic matter and nutrients via the ‘viral shunt’, and contributing to microbial niche differentiation through horizontal gene transfer [1–4]. In addition, some viruses encode “host genes” or auxiliary metabolic genes (AMGs) that metabolically reprogram their hosts during infection, including the well-documented cases of cyanobacterial viruses that express photosynthesis genes during infection and contribute significantly to marine microbial photosynthesis [5–7]. The extent of viral-encoded AMGs is just now being revealed, with detected AMGs so far also involved in C, N, P, and S metabolism [4, 8–12].

While viral ecology and the potential influences of viral-encoded AMGs has been examined in global-scale studies (e.g.,[10, 13]), there have been few investigations of viruses in marine oxygen minimum zones [11, 12, 14–16]. Marine oxygen minimum zones (OMZs) may be considered as ‘extreme environments’, but they constitute ~7% of oceanic volume and have increased substantially due to global climate change over the past fifty years, especially in the North and Equatorial Pacific where the volume of waters considered functionally anoxic (dissolved O_2_ below the detection limit [17]) have quadrupled [18, 19]. The expansion of OMZs is predicted to have positive feedbacks on climatologically active trace gases including CH_4_, N_2_O and DMS [20, 21] as a result of the chemotrophy performed by the unique microbial assemblages present in these regions [20]. Microbes in these areas deplete bioavailable nitrogen through anaerobic ammonium oxidation (anammox) and denitrification, accounting for 30–50% of oceanic nitrogen removal [21, 22], and also play roles in dissimilatory sulfur oxidation and sulfate reduction [23]. Recent research in OMZs has largely focused on the cycling of these major nutrients, as well as the taxonomically and functionally unique microbes responsible for key metabolic processes [20, 21].

Previous research has shown that oxygen is a driver of viral community structure in the Northeast Subarctic Pacific Ocean OMZ [24] and significantly affects viral infections of the ecologically important SUP05 bacteria in the Saanich Inlet OMZ [8]. Prior work to explicitly examine OMZ viral community structure using non-quantitative methods suggests that viral diversity is low in the Eastern Tropical South Pacific (ETSP) OMZ, but that OMZ viruses contain diverse metabolic genes that can affect biogeochemical cycling [14]. However, more recent work in the ETSP and Cariaco Basin has suggested that viral diversity remains high within the OMZ [15, 16]. Thus, there is critical need for quantitative datasets across diverse OMZs to provide the foundational understanding required to incorporate viruses into OMZ ecosystem models.

Here we investigated viral community structure and potential ecological impacts at two stations in the Eastern Tropical North Pacific (ETNP; Supplementary Fig. S1), which, located south of Baja California, encompasses 41% of global OMZ area and is the largest permanent OMZ [19]. To this end, we combined quantitative microscopic and metagenomic methods to evaluate the influence of environmental parameters, including oxygen concentrations, on (i) viral and bacterial abundances, (ii) lytic viral infection frequency in bacteria, (iii) viral community structure based on morphology and metagenomically-derived viral population abundances, and (iv) the distribution of viral-encoded AMGs. The results from this study indicate that while viral diversity and infection frequency remain high within the ETNP OMZ, the structure of the viral community and the composition of viral-encoded AMGs are substantially altered in the oxycline and the functionally anoxic core of the OMZ.

## Results and discussion

### Environmental conditions, viral and bacterial abundance, and frequency of infection

**Fig. 1:**
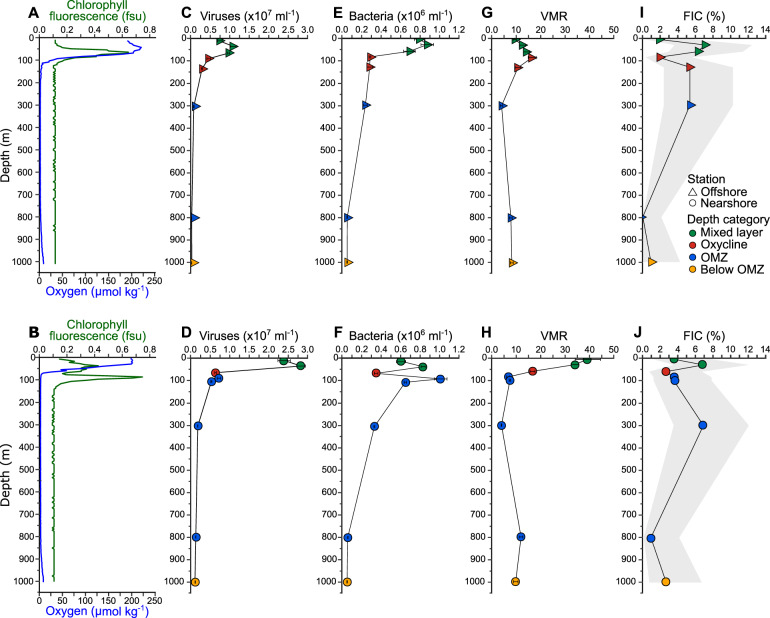
Depth profiles of environmental and microbial parameters. Oxygen concentration and chlorophyll fluorescence (**A**, **B**), viral (**C**, **D**) and bacterial concentrations (**E**, **F**), VMR (**G**, **H**), and FIC (**I**, **J**) at each station are displayed. Error bars represent standard deviations of the means of triplicate samples. Shaded areas in **I**–**J** represent positive and negative 95% confidence intervals for single samples.

Physiochemical parameters were measured using a Conductivity Temperature Depth profiler equipped with a fluorometer and dissolved oxygen sensor at two stations of the ETNP, one nearshore and one offshore. Examination of environmental conditions revealed similarities and key differences between the nearshore and offshore stations in the ETNP (Supplementary Fig. S1). While both stations exhibited a strong OMZ with oxygen concentrations below detection for ca. 700 m within the water column, the nearshore station revealed a shoaling of the oxycline relative to the offshore station (Fig. 1A, B). Temperature and oxygen profiles at both stations indicated the presence of a diurnal mixed layer modulated by air temperature and wind-driven mixing (Supplementary Fig. S2A, B). We thus use the term “mixed layer” as a depth category indicating the upper, oxygenated portion of the water column, distinct from the oxycline, OMZ, and below the OMZ. Phosphate and nitrate levels increased with depth beginning in the lower portion of the oxycline at both stations (Supplementary Fig. S2C–F) and there were noticeably higher peaks of nitrite and ammonium in the oxycline at the offshore station (Supplementary Fig. S2G–J).

Depth profiles of chlorophyll fluorescence also differed markedly between the stations, with a single peak at the offshore station, but two maxima at the nearshore station (Fig. 1A, B). This secondary chlorophyll maximum (SCM) within the OMZ of the nearshore station has been previously documented in both the ETNP [17] and Eastern Tropical South Pacific (ETSP) OMZs [25]. This oxygen-depleted portion of the water column maintains an active photosynthetic community, including *Synechococcus* and *Prochlorococcus* [26] that drive a cryptic oxygen cycle in which oxygen is consumed as fast as it is produced [25, 27].

Microbial abundances for each sample were quantified using epifluorescence microscopy. Viral and bacterial concentrations had local maxima in the upper, oxygenated portion of the water column, with positive correlations between them and with oxygen concentration (Fig. 1C–F, Supplementary Fig. S3). While some studies have reported secondary maxima of viral concentrations in marine and freshwater OMZs [14, 28, 29], this was not evident in the ETNP. There were notable differences between stations, including (i) approximately twice the concentration of viruses in the mixed layer at the nearshore station, resulting in a much higher virus-to-microbe ratio (VMR), and (ii) the presence of a secondary maximum in bacterial concentration at the nearshore SCM that was accompanied by only a minor increase in viral concentration (Fig. 1C–H).

In contrast to the VMR, the frequency of infected cells (FIC) as estimated using transmission electron microscopy was similar between stations, reaching local maxima in the mixed layer as well as the OMZ (Fig. 1I, J). VBR was thus not correlated with FIC (Supplementary Fig. S3), reflecting the complex balance between production of viruses and bacteria with removal of viruses through decay [30, 31] and mortality of bacteria resulting from numerous sources [32]. The increased FIC in the OMZ indicated that viruses were still lytically replicating in the OMZ as has been observed in multiple prior studies [28, 29, 33–35], resulting in a lack of correlation with oxygen concentration (Supplementary Fig. S3).

### Morphological diversity of viral communities

Viral morphology, including morphotype and capsid diameters, was analyzed using quantitative transmission electron microscopy [36]. We found non-tailed viruses to be the most abundant morphotype at both stations and all depths, ranging from 56–93% of each sample (Supplementary Fig. S4), consistent with a global survey in the upper water column [36]. The percent of non-tailed viruses was positively correlated with depth, and negatively with temperature and oxygen (Supplementary Fig. S3). This suggests either (i) an increase with depth of bona fide non-tailed dsDNA viruses such as the *Autolykiviridae*, which infect among others many widespread *Vibrio* species [37], (ii) an increased abundance of non-tailed ssDNA viruses [38], or (iii) a loss of tails as an initial step of natural tailed virus decay as has previously been suggested [36].

Viral capsid widths were also similar to those reported for the global upper oceans [36]. While viral capsid width did vary significantly among all samples (global ANOVA *p* < 0.001), there was not an evident trend in overall capsid widths with depth or between stations (Supplementary Fig. S5). However, capsid width distributions were significantly related to depth, temperature, oxygen, chlorophyll fluorescence, nitrite, and nitrate (*p* < 0.05, Supplementary Fig. S6A–I). This indicates that viral community structure in the ETNP, based on morphology, is driven directly or indirectly by environmental variables as has previously been shown [13, 36].

### Population-level diversity of viral communities

**Fig. 2:**
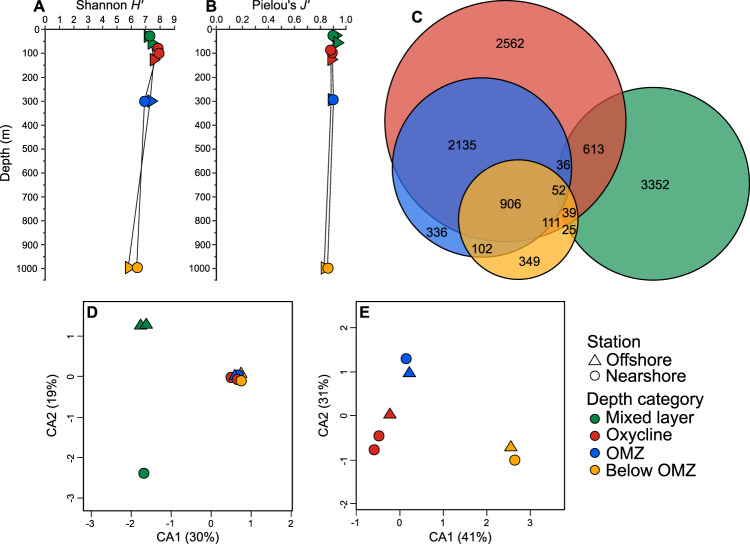
Diversity and distribution of viral populations in the ETNP. Shannon index *H′* (**A**) and Pielou’s evenness *J’* (**B**). The Euler diagram (**C**) shows the number of unique and shared viral populations in each group of samples for both stations combined (stress = 0.0011%). Correspondence analyses based on relative abundances of viral populations compare all samples (**D**) and the subset of samples from low-oxygen environments (**E**). For panels **D** and **E**, the percentage of inertia explained by CA1 and CA2 are reported on the axes.

Viral contigs were identified using VirSorter [39] and clustered into 10,601 populations at 95% ANI. Unsurprisingly, less than 1% of observed viral populations could be assigned a taxonomic identification (Supplementary Fig. S7) using RefSeq (version 74), as is common in marine viromes [40]. None of the fifty most abundant viral populations (12.2% of total abundance), which are mostly present in the low-oxygen samples at both stations, could be assigned a specific taxonomic identification. Of the less-abundant populations that could be identified, most were closely related to *Synechococcus* phages or other cyanophages, which have previously been identified in the mixed layer, oxycline, and even below the oxycline in the ETNP [26, 41]. Diversity (Shannon *H*’) and evenness (Pielou’s *J*’) of viral community structure based on relative population abundances in this study were similar to previous results from a global ocean survey [13], with a mean diversity and evenness of 7.12 and 0.899, respectively. Both viral diversity and evenness were consistent among all samples (Fig. 2A, B). This is in contrast to a previous study in the ETSP OMZ, which reported much lower viral alpha diversity and evenness, especially within the OMZ core [14]. However, that study used multiple displacement amplification to amplify nucleic acids for the metagenomes, which was later shown to have substantial biases (reviewed by [42, 43]). A recent study reported that diversity of free-living and particle-associated bacterial communities in the ETNP peaks near the SCM [44]. However, the slight increase in oxycline viral diversity we observed was not significant (Fig. 2A), indicating that extracellular viral community diversity patterns do not mirror those of the bacterial community.

For both stations combined, viral communities in the surface mixed layer were quite distinct from those in low-oxygen samples, with few shared populations (Fig. 2C, D). Most viral populations were unique to either the mixed layer or the oxycline (*n* = 5914), with populations in the OMZ core predominantly representing a subset of the oxycline community (Fig. 2C). Hierarchical clustering of the viral populations revealed two clusters comprised of the mixed layer communities and the low-oxygen communities, respectively, and their subtle differences in relative abundance of viral populations was visualized using a heatmap (Supplementary Fig. S8). Mixed layer communities showed clear differences between stations, as well as compared to low-oxygen communities (Fig. 2D), and all communities were significantly related to temperature, salinity, oxygen, and chlorophyll fluorescence (*p* < 0.05 for all; Supplementary Fig. S9A–I). After removal of the mixed layer communities, the low-oxygen subset of communities clustered by depth category rather than station (Fig. 2E) and were significantly influenced by depth, temperature, salinity, oxygen, chlorophyll fluorescence, nitrite and nitrate concentrations (*p* < 0.05 for all, Fig. S9J–R). These findings are similar to a previous investigation of viral communities in the subarctic North Pacific OMZ that showed significant differences between photic and aphotic samples, though distance from shore was less influential [24]. In the ETNP, we suspect that coastal upwelling accounted for the differences between mixed layer samples [45], while the strong influences of oxygen and depth overwhelm factors that differ between our stations in low-oxygen samples.

### General AMG trends

**Fig. 3:**
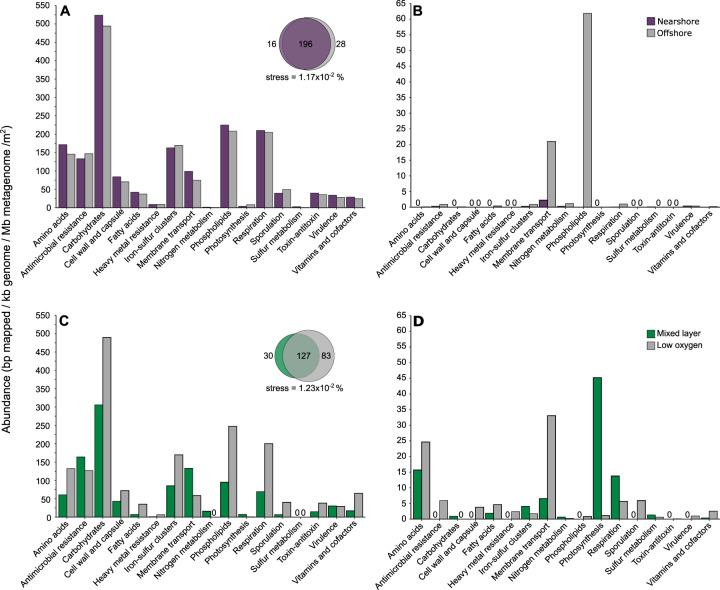
Depth-integrated relative abundances of AMGs by category. Those found at both stations (**A**), unique to one station (**B**), found in both the mixed layer and low-oxygen environments (**C**), or unique to either the mixed layer or low oxygen environments (**D**) are displayed. Euler diagram insets in panels **A** and **C** represent the number of unique and shared PFAMs by station (within panel **A**) and depth category (within panel **C**).

Viral-encoded AMGs can metabolically reprogram their hosts during infection and significantly impact biogeochemical cycling, even accelerating host niche differentiation [10, 46–48]. While most of the genes that could be assigned functions within the ETNP viral metagenomes were related to viral replication, including DNA replication and repair and viral structural proteins, there were 247 genes annotatable as Class I AMGs, and 81 as Class II AMGs (3% of total genes; Supplementary Fig. S10). As with prior studies (reviewed by Roux et al. [10, 41]), we found that AMGs in the ETNP viral community were related to environmental conditions. The majority of AMGs were present at both the offshore and nearshore stations at similar abundances (Fig. 3A). Of the few AMGs that were unique to one station, the most abundant were related to membrane transport and phospholipid metabolism at the offshore station (Fig. 3B). This distribution of AMGs was consistent with the population-level structure of the viral community in which the samples below the mixed layer were highly similar between stations (Fig. 2D, E).

While 52% of the AMGs were present in both mixed layer and low-oxygen samples, the functional categories of these shared AMGs were present at different abundances within depth categories (Fig. 3C). Eleven shared gene categories were more abundant in low-oxygen samples, including amino acid metabolism, carbohydrate metabolism, cell wall and capsule formation, and sporulation genes (Fig. 3C). Of the AMGs unique to the mixed layer, most were related to photosynthesis, respiration, and amino acid metabolism, while those unique to low-oxygen samples were primarily related to amino acid metabolism and membrane transport (Fig. 3D). These larger differences in AMG composition when comparing mixed layer versus low-oxygen samples again support the population-level comparisons of the viral communities (Fig. 2D), in which oxygen concentration is a major driver of viral community composition (Supplementary Fig. S9E).

**Fig. 4:**
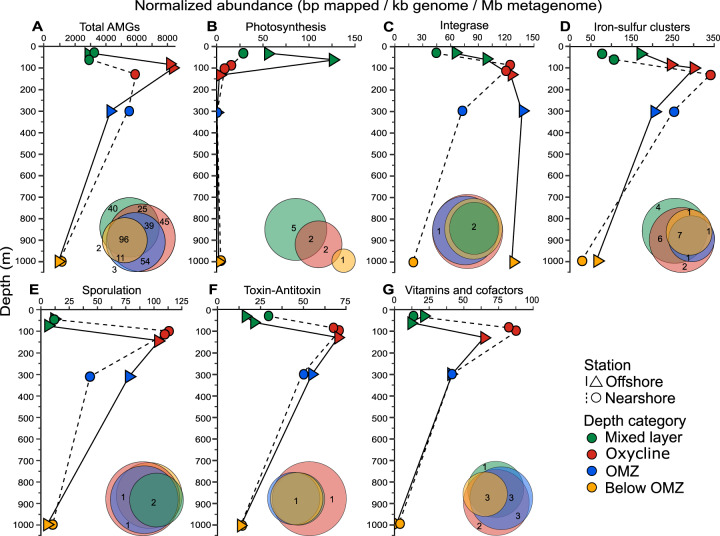
Depth profiles of relative abundances of select AMG categories detected in this study. All detected viral AMGs (**A**) and selected AMG categories (**B**–**G**) are displayed. Euler diagram insets represent the number of PFAMs unique and shared by depth category.

To further investigate the distribution of AMGs, we examined depth profiles of AMG functional categories at each station (Fig. 4A–G and Supplementary Fig. S11A–L). All AMGs combined exhibited relatively low abundance in the mixed layer at both stations, increased dramatically in the oxycline, and then decreased with depth (Fig. 4A). The number of unique and shared AMGs among depth categories (Fig. 4A, inset) also reflected overall community structure (Fig. 2C), with the majority of AMGs shared across all depth categories, and the most unique AMGs found within the mixed layer and oxycline. This indicates that viruses do not utilize many unique AMGs among the depth categories, but instead that commonly occurring ones are selectively enriched in specific sections of the water column as described below.

### Viral influences on photosynthesis, nitrogen, and sulfur metabolism

AMGs related to photosynthesis were unsurprisingly most abundant in the mixed layer (Fig. 4B), where they function to enhance host photosynthetic activity (reviewed by Aldunate et al. [41]). We also observed extremely low abundances of photosynthesis-related AMGs below the OMZ as has been reported in the subarctic North Pacific OMZ, likely due to phage released from sinking organic particles at depth [11]. The most abundant photosynthesis AMGs in the ETNP, *psbA/D* and *psbN*, encode for photosynthetic reaction center proteins and have been observed in oceanic datasets previously [11, 14, 49, 50] as they are widely distributed within marine cyanophage isolates. However, while the abundance of these photosynthesis genes was significantly positively correlated with oxygen and chlorophyll concentrations (Supplementary Fig. S12), they were much less abundant at the nearshore station than expected given the high chlorophyll concentration present in the mixed layer and SCM (Fig. 1A, B). Their low abundance at the nearshore station SCM may be due to (i) a high prevalence of uncharacterized viruses that infect the unique cyanobacterial ecotypes in the SCM as found in the ETSP [25, 26], which may not contain these core photosynthesis genes, (ii) a selection for viruses that do not contain these genes, as they may only impart a selective advantage under high light conditions [51, 52], or (iii) a counter selection of these genes because the viruses have short latent periods during which the viral genes could not be sufficiently expressed to boost host photosynthetic potential, as has been suggested with some cyanobacterial isolates previously [50, 52].

Recent work regarding viruses in the ETSP and Cariaco basin has suggested viral AMGs may influence nitrogen and sulfur cycling within the low-oxygen layer [12, 16]. Although we detected some AMGs related to these processes within the ETNP, they were present at low abundances. Depth profiles of nitrogen metabolism AMGs revealed a peak in the mixed layer and oxycline at the nearshore and offshore stations, respectively (Supplementary Fig. S11H), then decreasing with depth. Sulfur metabolism AMGs were most abundant in the mixed layer at both stations and decreased with depth, though the nearshore station remained slightly higher abundance until below the OMZ (Supplementary Fig. S11K). We detected no genes associated with the processes that dominate nitrogen cycling in the ETNP, including annamox, nitrite oxidation, denitrification, and ammonia oxidation [44, 53–55]. We detected *draT*, which is part of a system that inhibits nitrogenase under high-ammonium conditions or energy depletion [56], though this gene was present at very low levels only in the OMZ core of the offshore station. Additionally, *nifU* was present in all samples, though as it is often found in organisms not capable of fixing nitrogen [57, 58], we categorized it as an AMG related to iron–sulfur cluster assembly. The most abundant sulfur metabolism gene we detected was *tauD*, which catalyzes the oxygenolytic release of sulfite from taurine [59] and was present in the mixed layer and oxycline of both stations. *SoxY*, which encodes part of a thiosulfate oxidizing enzyme complex [60], was also observed in this study at very low abundance in the oxycline and OMZ core of both stations. Sulfur metabolism AMGs previously observed in marine viromes, namely *dsrC*, *soxB*, and *rdsrA*, were not observed here [8, 61–63].

Although the ETNP is known to contain a near-complete nitrogen cycle [21, 54, 55, 64] and our chemical profiles indicated the presence of these processes (Supplementary Fig. S2E–J), as well as sulfur oxidation and sulfate reduction [63], we detected very few AMGs related to these processes. This seemingly introduces a paradox in which viral-encoded AMGs do not include genes related to the dominant nitrogen and sulfur cycling processes in the ETNP. However, these results are parsimonious with the hypothesis that viruses will only contain AMGs required to enhance rate-limiting steps in reactions beyond that of which the host requires in order to increase viral replication [10, 47]. Thus, we suggest that AMGs related to nitrogen and sulfur metabolism in the ETNP are so low in abundance because host metabolic processing of these nutrients is sufficient to sustain requirements for viral replication. Our results indicate that viruses influence host physiological state in the ETNP more than manipulate metabolic reactions.

### Viral replication strategy and influences on host physiological state

We found that phage integrases increased in relative abundance with depth at the offshore station, while at the nearshore station they peaked in the oxycline and decreased below it (Fig. 4C). This suggests that a higher portion of the extracellular viruses at the offshore station seem to have the ability to integrate into their hosts as lysogens, which has been suggested to increase with depth and environmental stress [29, 65]. The increased potential for lysogeny with elevated stress is consistent with the patterns regarding viral-encoded genes related to host stress described below.

Iron–sulfur cluster synthesis, sporulation, toxin-antitoxin (TA), and antimicrobial resistance (AMR) genes were detected throughout the ETNP. Depth profiles revealed that all three AMG categories were most abundant in the oxycline at both stations and decreased in abundance with depth (Fig. 4D–F), resulting in no correlations with environmental variables (Supplementary Fig. S12). Iron–sulfur clusters are extremely important prosthetic groups required by many microbial enzymes central to metabolism [58, 66–69], including electron transfer and modulation of gene expression under oxidative and redox stress conditions [67, 70]. The most abundant of these AMGs we detected were involved in cluster assembly or biosynthesis, several of which have been observed in previous viromes [11, 71]. One such assembly gene, *sufE*, has been shown to enhance the activity of *sufS* to assemble housekeeping iron–sulfur clusters under oxidative stress [72–76], but cannot function alone [74], so its presence without *sufS* in the ETNP suggests that host production of *sufE* is the limiting step in this iron–sulfur cluster formation. The other most common iron–sulfur cluster biosynthesis pathways are the *isc* [58, 66, 77] and *nif* [57, 58] operons: of these, we detected *nifU* as mentioned above. Additionally, we detected several hits to iron–sulfur cluster binding domains commonly found in ferredoxins that mediate electron transfer in various metabolic reactions [78]. The presence of these iron–sulfur cluster genes suggests that viruses may be boosting host response to oxidative and redox stress, especially in the oxycline.

We detected several AMGs related to sporulation, a bacterial survival strategy where the cell produces a hardy endospore form that can persist for extended periods of time [79]. These genes were most abundant in the oxycline and decreased with depth below it, with the offshore remaining more abundant than the nearshore in the OMZ core (Fig. 4E). The most abundant of these, *spoVR*, along with *spoVS*, *spoIIE*, and *spoVG*, are related to the formation of bacterial spores [80]; of these, *spoVS* has been seen in soil viromes previously [9]. Phages have been shown to persist in bacterial spores, and some lysogenic phages of *Clostridium botulinum* and *Bacillus* species have been shown to encode key virulence genes as well as sporulation genes to modulate host metabolism [81–84]. Though many spore-producing microbes are obligate or facultative anaerobes, the unique stressors of the unstable oxycline environment may limit these hosts. Thus, ETNP viruses may then help induce sporulation in their hosts to persist similarly to previously characterized phages of *Clostridium* and *Bacillus* species [83, 85].

We found that the distribution of AMGs related to TA systems was similar to that of sporulation-related genes, though there was little difference between stations (Fig. 4F). The main driver of this pattern was a gene that encodes zeta toxin, a well-characterized plasmid-borne postsegregational killing system [86, 87]. Some temperate phages that persist as extrachromosomal prophages or integrate into host genomes use genes for plasmid inheritance and persistence, including toxin-antitoxin genes [65], though we did not identify any integrase genes on populations containing TA AMGs. A gene for the SymE toxin, part of a type I TA system that is often found in bacterial chromosomes [88], was present at much lower abundance. In the oxycline, host cells under extreme environmental stress may be more likely to expel plasmids or extrachromosomal prophages because maintenance of them is costly [89–91]. We suggest that postsegregational killing systems such as the zeta toxin can deter hosts from expelling viruses while under the unique stressors of the oxycline, allowing the viruses to lyse their hosts.

AMGs related to AMR and virulence peaked in the oxycline at the nearshore station and within the oxycline and OMZ core offshore (Supplementary Fig. S11B, L), resulting in negative and positive correlations with depth and temperature, respectively (Supplementary Fig. S12). Viruses have been shown to help defend their hosts from other microbes in free-living and biofilm settings, as well as influence their bacterial host’s ability to infect their own multicellular hosts and/or establish productive infection [65, 84, 87, 92, 93]. Two of the most abundant AMGs related to AMR and virulence in our dataset are related to the production of alginate, which helps form the capsule of *P. aeruginosa* that protects the bacterium from antibiotics and is important in biofilm production [94]. Other abundant AMGs include *tylF* and a tryptophan halogenase, which are involved in the production pathways of a macrolide that functions against Gram positive bacteria, and the broad-spectrum anti-fungal pyrrolnitrin, respectively [95, 96]. The most abundant virulence gene, a glycosyltransferase involved in the biosynthesis of lipopolysaccharide, contains an endotoxin in some Gram-negative bacteria including *Pseudomonas* [97–99]. Boosting the production of antimicrobial compounds and virulence factors in their hosts could allow viruses in the ETNP to keep the hosts alive long enough to facilitate viral replication. Overall, the presence of these genes related to host physiological state is once again parsimonious with the hypothesis that viruses will only contain AMGs required to enhance rate-limiting steps in reactions beyond that of which the host requires in order to increase viral replication [10, 47]. In the ETNP, the survivability of bacterial hosts under stress due to varying nutrient and oxygen conditions may be a more important factor in viral success than speeding up energy or carbon production.

### AMGs related to vitamin metabolism

Vitamin and cofactor metabolism AMGs exhibited relatively low abundance in the mixed layer, increased dramatically in the oxycline, and then decreased with depth at both stations (Fig. 4G), resulting in no correlations with environmental variables (Supplementary Fig. S12). Several of these AMGs, including the most abundant pantoate transferase, are involved in the biosynthesis of pantothenate (vitamin B_5_), which is required for the synthesis of coenzyme A (CoA, [91, 92]), or are directly involved in CoA synthesis [100, 101]. CoA is an essential coenzyme that plays a key role in fatty acid metabolism and the biosynthesis of peptides [100]; notably, we detected a pantothenate kinase, which is the rate-limiting step in CoA biosynthesis, in all samples except below the OMZ. This suggests that viruses in the ETNP may be boosting pantothenate and CoA levels to bolster host metabolism. We also observed *cobT*, related to the aerobic production of cobalamin in bacteria [102], mostly in the oxycline of both stations. Recent research suggests the importance of cobalamin-producing archaea, especially *Thaumarchaeota* [103], in the ETNP as potential drivers of community structure as this highly-sought after coenzyme is produced by few organisms [104, 105], giving them a selective advantage. A study investigating putative archaeal viruses in the ETNP OMZ region did not identify any viruses with cobalamin biosynthesis genes, including *cobT*, but this may be due to the conservative nature of the analysis [105]. Thus, viruses in the ETNP may be modulating community structure by boosting cobalamin production in their hosts, especially in the oxycline at both stations and in deep offshore areas.

## Conclusions

**Fig. 5:**
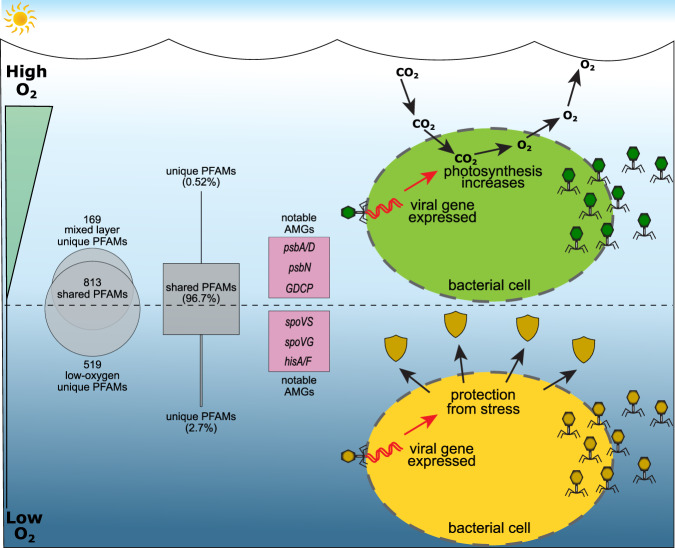
Diagram of key differences between surface and low-oxygen samples detected in this study. Total identified PFAMs, represented by the Euler diagram, were mostly ‘core’. These also represent most of the total normalized abundance of identified PFAMs, as represented by the boxes. Of those AMGs that were predominantly found in the mixed layer samples, the most abundant were *psbA/D* and *psbN*, which are involved in photosynthesis, and *GDCP* which is involved in glycine synthesis. The most abundant core low-oxygen AMGs include *spoVS* and *spoVG*, involved in sporulation, and *hisA/F* which is involved in histidine synthesis. This supports the hypothesis that the dominant viral strategy in the ETNP surface mixed layer is to boost host metabolism to facilitate viral replication, whereas those in the oxycline and OMZ are more likely to facilitate host persistence long enough for viral replication to occur.

Marine OMZs constitute a relatively large portion of the world’s oceans and harbor unique microbial communities that perform globally-important biochemical processes (reviewed by Paulmier et al. [19–21, 27]), yet information regarding the viral communities within these regions remains sparse. Here we demonstrate that there are some similarities, as well as substantial differences, in viral community ecological metrics throughout the water column in the ETNP OMZ region. While viral abundance decreased within the low-oxygen portion of the water column, the frequency of infected cells was approximately equivalent to that found in the upper-ocean mixed layer, indicating that viruses are both present and actively infecting bacteria within the OMZ. We also demonstrate that the diversity of the viral communities was consistently high throughout the water column, but that viral community population structure diverged significantly with oxygen concentration. Given the divergent microbially-driven biogeochemical cycling that occurs as a function of oxygen concentration in marine OMZs, and the importance of AMGs as drivers of viral population structure [4, 8–11], we expected that viral-encoded AMGs involved in nitrogen and sulfur cycling would drive the differences observed in viral community structure throughout the water column in the ETNP. Instead, we observed that while a high portion of annotated PFAMs were shared among the depth categories, their relative abundances indicate strong selection for different viral-encoded AMGs with varying oxygen concentration that are not related to nitrogen or sulfur cycling (Fig. 5). Our study shows that the AMGs enriched in the upper oxygenated water column are related to increasing host metabolism, including photosynthesis, while viral AMGs within the low-oxygen portion of the water column are primarily related to enhancing the host bacterium’s ability to deal with oxygen-related stress (Fig. 5). We reason that this is due to the requirement of viruses to streamline their genomes and retain only essential genes, as they are limited by physical space in their capsids [106] and the energy and materials required for DNA replication [107]. We speculate that there may only be selective pressure to maintain AMGs within viral populations under certain conditions, such as the presence of nitrogen-related metabolism genes found only within a certain range of nitrogenous nutrient concentrations [10]. Thus, when host metabolism is sufficient to support viral replication, viruses may have no need to encode AMGs related to the predominant biogeochemical reactions being performed by their hosts in that environment, but will instead selectively encode any gene that increases viral replication. In the ETNP OMZ region, our study indicates that viral influences are not predominantly related to directly altering biogeochemical reactions, but instead they encode genes that enhance their bacterial hosts’ ability to sufficiently maintain metabolic functions for the virus to replicate.

## Methods

### Sample collection

Samples were collected from the ETNP OMZ during the OMZ Microbial Biogeochemistry Expedition cruise (*R/V New Horizon*, 13–28 June 2013) as previously described [105]. Samples were collected from two stations: Station 6 was located on the continental shelf (18°55’12” N and 104°53’24” W), and Station 2 was located ~450 km west of Station 6 (18 °55’12” N and 108°47’60” W). We thus use the terms “nearshore” and “offshore” to describe Stations 6 and 2, respectively. At each station, water samples were collected from eight depths spanning the mixed layer (5 m, 30 m, 60 m at the offshore station; 5 m and 30 m at nearshore station), oxycline (85 m and 130 m at offshore station: 60 m, 85 m, 100 m at nearshore station), OMZ core (300 m and 800 m at both stations), and below the OMZ (1000 m at both stations). Seawater was collected using Niskin bottles on a rosette containing a Conductivity Temperature Depth profiler (Sea-Bird SBE 911plus, Sea-Bird Electronics Inc., Bellevue, WA, USA) equipped with a Seapoint fluorometer (Seapoint Sensors Inc., Exeter, NH, USA) and SBE43- dissolved oxygen sensor (Sea-Bird Electronics Inc., Bellevue, WA, USA). CTD profiles from this study were deposited at the Biological & Chemical Oceanography Data Management Office (BCO-DMO) [108].

### Microbial abundances

Triplicate samples (4 ml) for viral and bacterial enumeration were preserved with EM-grade glutaraldehyde (2% final concentration), flash-frozen in liquid nitrogen and stored between –72 °C and −80 °C until analysis. Viral and bacterial concentrations were determined using a previously described method [109] in which thawed samples were filtered onto 0.02-μm- pore-size filters (Anodisc, Whatman, GE Healthcare Life Sciences, Piscataway, NJ, USA), stained with SYBR Gold nucleic acid stain (Invitrogen, Life Technologies, Carlsbad, CA, USA) and enumerated using an epifluorescence microscope (Axio Imager. D2, Zeiss, Jena, Germany). Microbial abundances determined in this study were deposited at the BCO-DMO [110].

### Frequency of infected cells

The percentage of cells with active lytic viral infections was determined by transmission electron microscopy to quantify the frequency of visibly infected cells [31] using intact cells [35]. Samples preserved with EM-grade glutaraldehyde (2% final concentration) were flash-frozen and stored at –80 °C until analysis. Samples (12 ml) were centrifuged onto 200-mesh copper grids with carbon-stabilized formvar support (Ted Pella, Redding, CA, USA) made hydrophilic with 20 s of glow discharge with a sputter coater (Hummer 6.2, Anatech, Battle Creek, MI, USA) for 1 h at 55,000 × *g* using an ultracentrifuge (LM-80, Beckman Coulter, Brea, CA, USA) with a swing-bucket rotor. Grids were then stained with 0.5% uranyl acetate and analyzed as previously described [35] to determine the frequency of visibly infected cells using a transmission electron microscope (FEI Tecnai Spirit). The frequency of infected cells was then calculated from the frequency of visibly infected cells [111]. Frequency of infected cells data generated from this study were deposited at BCO-DMO [112].

### Morphological analysis of viral communities

Viral capsid diameters, tail length, and morphotype were determined using the qTEM method described previously [36]. Samples preserved with EM-grade glutaraldehyde (2% final concentration) were flash-frozen and stored at –80 °C until analysis. Viruses were deposited onto TEM grids using an air-driven ultracentrifuge (Airfuge CLS, Beckman Coulter, Brea, CA, USA), followed by positive staining with 2% uranyl acetate (Ted Pella, Redding, CA, USA). TEM grids were dried at room temperature overnight then stored desiccated until analysis using a transmission electron microscope (Philips CM12 FEI, Hilsboro, OR, USA) with 100 kV accelerating voltage. Micrographs of 100 viruses were obtained per sample using a MacroFire Monochrome charge-coupled device camera (Optronics, Goleta, CA, USA) and analyzed using ImageJ software (U.S. National Institutes of Health, Bethesda, MD, USA) [113] to measure the capsid diameter and tail length, and classify their morphotype as previously described [36]. Morphological data generated in this study were deposited at BCO-DMO [114].

### Metagenome preparation and analysis

Ten samples were used for metagenomics (30 m, 85 m, 100 m, 300 m, and 1000 m at the nearshore station: 30 m, 60 m, 130 m, 300 m, and 1000 m at the offshore station). For each sample, 20 liters of seawater were filtered through a 0.22 μm-pore-size filter (Steripak; Millipore Sigma, Burlington, MA, USA) and viruses in the filtrate were concentrated using iron chloride flocculation as previously described [115, 116] followed by storage at 4 °C. Viral particles were then resuspended using an ascorbic EDTA buffer and their DNA extracted using a Wizard DNA purification system (Promega, Fitchburg, WI, USA) after treatment with DNase I as previously described [105]. Extracted DNA was then sheared with a Covaris ultra-sonicator (Woburn, MA, USA), gel-purified to select fragments of 160–180 bp in length, and then ligated and amplified using the standard Illumina protocol with PCR amplification of the library. Sequencing was carried out on a HiSEq 2000 system at the DOE Joint Genome Institute (Berkeley, CA, USA), except for the 300 m sample from the offshore station which was sequenced at the University of Arizona Genome Center. Metagenome accession numbers and metadata were deposited at BCO-DMO [117].

Sequencing reads were quality trimmed to remove bases with quality scores lower than two standard deviations from the average score (across sequencing cycles), and bases with a quality score lower than 20. Reads ≥95 bp were assembled using Idba_ud v1.1.2 with default parameters [118]. Assembled contigs were analyzed using the program VirSorter v1.0.2 with the ‘--virome’ option [39], and all contigs ≥10 kb of categories 1 and 2 as well as manually curated contigs and prophage predictions of categories 3–6 were selected for further analysis. Selected viral contigs were clustered with nucmer 3 [119] at ≥95% ANI across ≥80% of their lengths, as in [13], to generate a pool of non-redundant ‘population contigs’. Information about the metagenome assemblies and VirSorter contigs can be found in Table S1.

Taxonomic annotation of the viral populations was determined based on affiliation of >50% of the genes with a reference genome from RefSeq (version 74; using a BLASTp comparison with thresholds of 50 for bitscore and 10^−5^ for e value). All viral populations generated as described above were used in subsequent analyses regardless of their taxonomic annotation. Functional annotations of all predicted proteins from ETNP viral contigs was based on a comparison to the PFAM domain database v.27 [120] with HmmSearch [121] thresholds of 30 for bit score and 10^−3^ for e value). PFAMs hits were manually categorized by their general function, including “DNA replication, recombination, repair; nucleotide metabolism”, “lysis”, “metabolism”, “structural”, and “transcription, translation, protein synthesis”. Of these, 270 could not be assigned to a functional category but did match a PFAM domain, whereas 452 did not match any PFAM domain (12.4% and 11.4% bp mapped/kb genome/Mb metagenome, respectively). We included Class I or II AMGs in our analyses based on the established definitions [11], resulting in 329 identified AMGs (16.1% bp mapped/kb genome/Mb metagenome). Of these, 81 could only be identified as peripherally associated with metabolism (8.0% bp mapped/kb genome/Mb metagenome).

### Statistical analyses

All statistical analyses were conducted using R version 3.5.1 [122]. Depth profiles, bar plots, stacked bar plots, and the heatmap were created using the ggplot2 package [123]. Shannon index (*H’*) and Pielou’s evenness (*J’*) were calculated using ‘diversity’ function in the vegan package [124]. Euler diagrams were visualized using the ‘venneuler’ function in the venneuler package [125].

Correlations were performed using the ‘rcorr’ function of the Hmisc package [126] using Pearson coefficients, and visualized using the ‘corrplot’ function of the corrplot package with an α of *p* < 0.05 [127]. We used a binomial test to compare the number of significant correlations we identified to the expected false-positive rate as previously described [128]. Briefly, we compared the expected false-positive rate of the number (*n*) of tests at our significance level α (*n* x α) to our number of significant correlations (‘successes’ under a binomial distribution). Because our number of determined significant correlations far exceeded the false-positive rate, we considered the correlations to be significant.

Correspondence analysis was performed using the ‘cca’ function in the vegan package [124] to obtain an ordination plot of viral communities based on viral capsid diameters or populations for each sample as in [36]. Average optimal capsid diameter bin size was determined using the method of [129]. Vectors and response surfaces of environmental variables were fitted to the CA ordination plot using the function ‘envfit’ in vegan with 10,000 simulations to estimate *p* values and the function ‘ordisurf’ in vegan, respectively [124]. Hierarchical clustering was performed using the ‘pvclust’ function of the pvclust package [130] with 100 bootstrap replications to estimate *p* values.

## Supplementary information

Supplemental Captions

Figure S1

Figure S2

Figure S3

Figure S4

Figure S5

Figure S6

Figure S7

Figure S8

Figure S9

Figure S9

Figure S10

Figure S11

Figure S12

Table S1
