# Supplementary material for: Viral community analysis in a marine oxygen minimum zone indicates increased potential for viral manipulation of microbial physiological state
Source: ISME J. 2021 Nov 6;16(4):972–82. doi: 10.1038/s41396-021-01143-1 (PMC8940887; doi:10.1038/s41396-021-01143-1)
Supplement: Supplementary file 1 — Supplemental Captions [file 41396_2021_1143_MOESM1_ESM.docx]

Viral community analysis in a marine oxygen minimum zone indicates increased potential for viral manipulation of microbial physiological state

**Supplementary Captions**

**Figure S1.** Location of the offshore and nearshore stations in the ETNP for this study. Image generated with Ocean Data View (Schlitzer R. 2016. Available from: https://odv.awi.de/).

**Figure S2.** Depth profiles of temperature (A-B) and concentrations of phosphate (C-D), nitrate (E-F), nitrite (G-H), and ammonium (I-J) at each station.

**Figure S3.** Matrix of Pearson correlations between environmental variables, viral and bacterial concentrations, VBR, relative abundances of viral morphotypes, and FIC. Colors represent Pearson correlation coefficients. * indicates significant correlations (p < 0.05).

**Figure S4.** Relative abundance of viral morphotypes in all samples (n = 100 for each sample).

**Figure S5.** Violin plots comparing viral capsid diameter distributions in depth categories at each station. Left, middle, and right vertical lines in each plot correspond to the 25^th^, 50^th^ (median), and 75^th^ percentiles, respectively. Points represent outliers (> 2 standard deviations above the median for each sample). The dotted vertical line shows the global median capsid diameter for the entire dataset. Letters indicate significant differences between categories (ANOVA with Tukey’s post-hoc test, *p* < 0.001 for all). The number of viruses included within each plot is given in parentheses.

**Figure S6.** Correspondence analysis based on the distribution of viral capsid diameters with 5 nm bins for all samples (A). Response surfaces are shown for all environmental variables (B – I). *P* values highlighted in red are significant (*p* < 0.05). The percentage of inertia explained by CA1 and CA2 are reported on the axes.

**Figure S7.** Figure S7. Taxonomic identification of all viral populations (smaller pie graph) and those that were taxonomically annotated (larger pie graph) for both stations. Those that made up

<2% of each category are combined as "other". The area of each pie chart represents the

relative abundance of each taxon, and the numbers inscribed in each section show the

number of affiliated viral populations.

**Figure S8.** Heatmap based on normalized relative abundances of viral populations. Rows represent samples, labelled by station, depth category, and depth. Columns represent viral populations with the normalized relative abundance (log10 transformed) shown in purple. Hierarchical clustering of samples represented by the dendrogram revealed two clusters, denoted by the dotted line. Numbers on dendrogram nodes represent approximately unbiased bootstrapping values, based on 100 permutations.

**Figure S9.** Correspondence analysis based on the relative abundance of viral populations for all samples (A) and the subset of low-oxygen samples (J). Response surfaces are shown for each environmental variable for all samples (B – I) and the sample subset (K – R). ­­­­*P* values highlighted in red are significant (*p* < 0.05). The percentage of inertia explained by CA1 and CA2 are reported on the axes.

**Figure S10.** Relative abundance of all PFAM categories detected within viral populations in this study. “No known function” indicates predicted genes with hits to the KEGG database, but whose function has not been determined experimentally.

**Figure S11.** Depth profiles of additional AMG categories. Euler diagram insets represent the number of PFAMs unique and shared by depth category.

**Figure S12.** Matrix of Pearson correlations between environmental variables and AMG categories. Colors represent Pearson correlation coefficients. * indicate significant correlations (*p* < 0.05).

**Table S1.** List of viromes included in this study. Station number, depth, and sequencing effort are indicated for each virome sample.
