# Supplementary material for: Viral community analysis in a marine oxygen minimum zone indicates increased potential for viral manipulation of microbial physiological state
Source: ISME J. 2021 Nov 6;16(4):972–82. doi: 10.1038/s41396-021-01143-1 (PMC8940887; doi:10.1038/s41396-021-01143-1)
Supplement: Supplementary file 2 — Figure S1 [file 41396_2021_1143_MOESM2_ESM.pdf]

Fig. S1

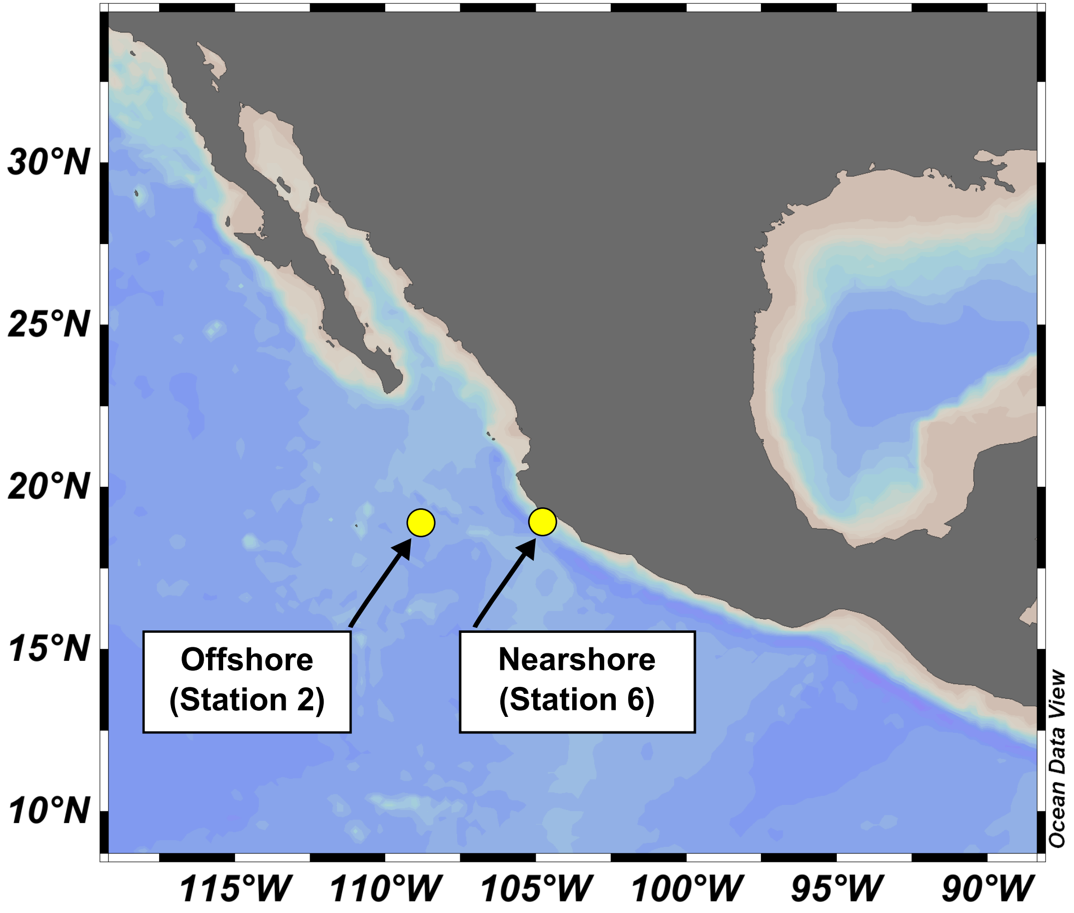

Figure S1. Location of the offshore and nearshore stations in the ETNP for this study. Imagegenerated with Ocean Data View (Schlitzer R. 2016. Available from: https://odv.awi.de/).
