# Supplementary material for: Viral community analysis in a marine oxygen minimum zone indicates increased potential for viral manipulation of microbial physiological state
Source: ISME J. 2021 Nov 6;16(4):972–82. doi: 10.1038/s41396-021-01143-1 (PMC8940887; doi:10.1038/s41396-021-01143-1)
Supplement: Supplementary file 3 — Figure S2 [file 41396_2021_1143_MOESM3_ESM.pdf]

Fig. S2

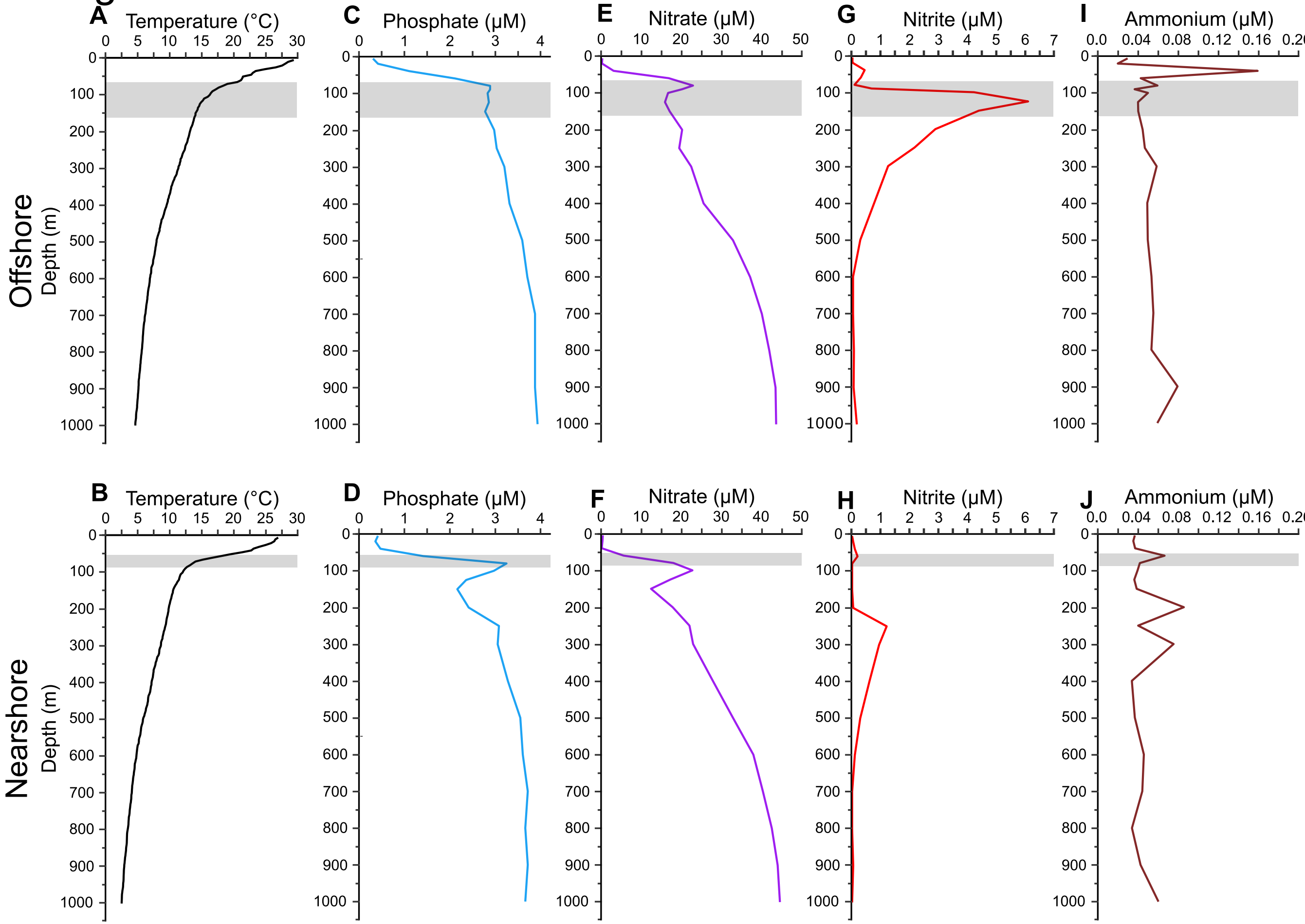

Figure S2. Depth profiles of temperature (A-B) and concentrations of phosphate (C-D), nitrate (E-F), nitrite (G-H), and ammonium (I-J) at each station. Gray box on each panel represents the oxycline.
