# Supplementary material for: Viral community analysis in a marine oxygen minimum zone indicates increased potential for viral manipulation of microbial physiological state
Source: ISME J. 2021 Nov 6;16(4):972–82. doi: 10.1038/s41396-021-01143-1 (PMC8940887; doi:10.1038/s41396-021-01143-1)
Supplement: Supplementary file 4 — Figure S3 [file 41396_2021_1143_MOESM4_ESM.pdf]

Fig. S3

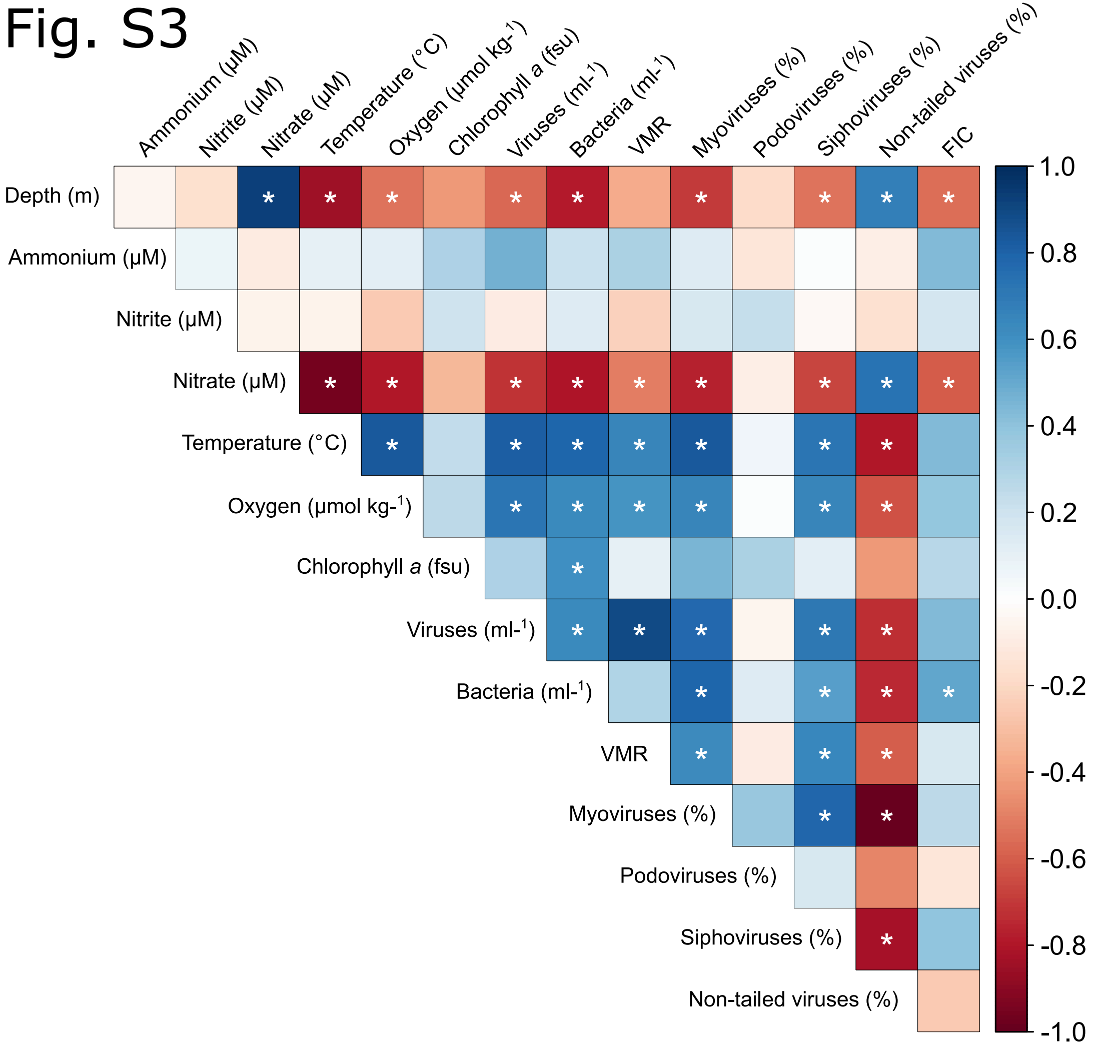

Figure S3. Matrix of Pearson correlations between environmental variables, viral and bacterial concentrations, VBR, relative abundances of viral morphotypes, and FIC. Colors represent Pearson correlation coefficients. * indicates significant correlations ($p < 0.05$).
