# Supplementary figures and images for: Viral community analysis in a marine oxygen minimum zone indicates increased potential for viral manipulation of microbial physiological state
Source: ISME J. 2021 Nov 6;16(4):972–82. doi: 10.1038/s41396-021-01143-1 (PMC8940887; doi:10.1038/s41396-021-01143-1)

Fig. S4

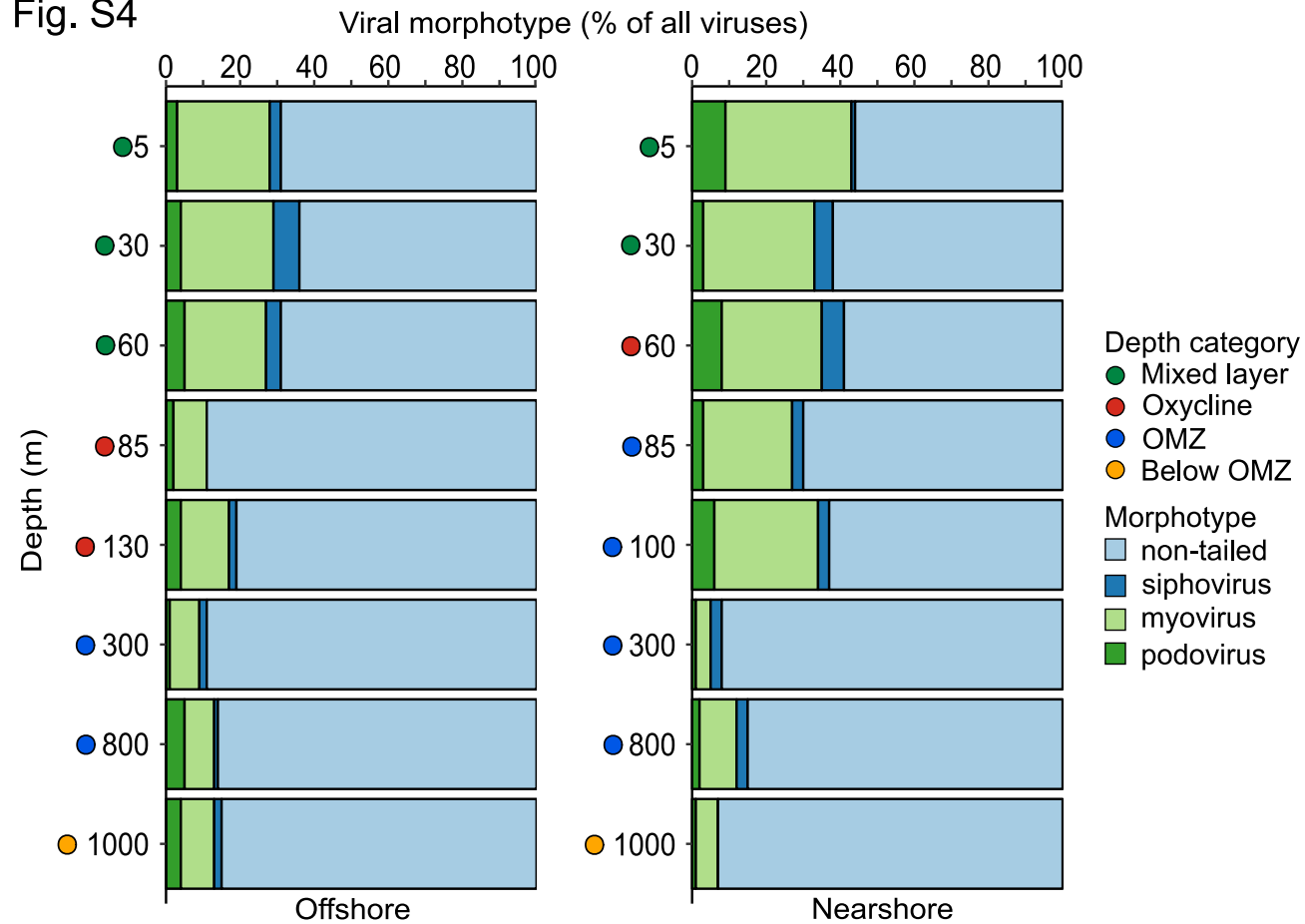

**Figure S4.** Relative abundance of viral morphotypes in all samples (n = 100 for each sample).

Supplement: Supplementary file 5 — Figure S4 [file 41396_2021_1143_MOESM5_ESM.pdf]

Fig. S9

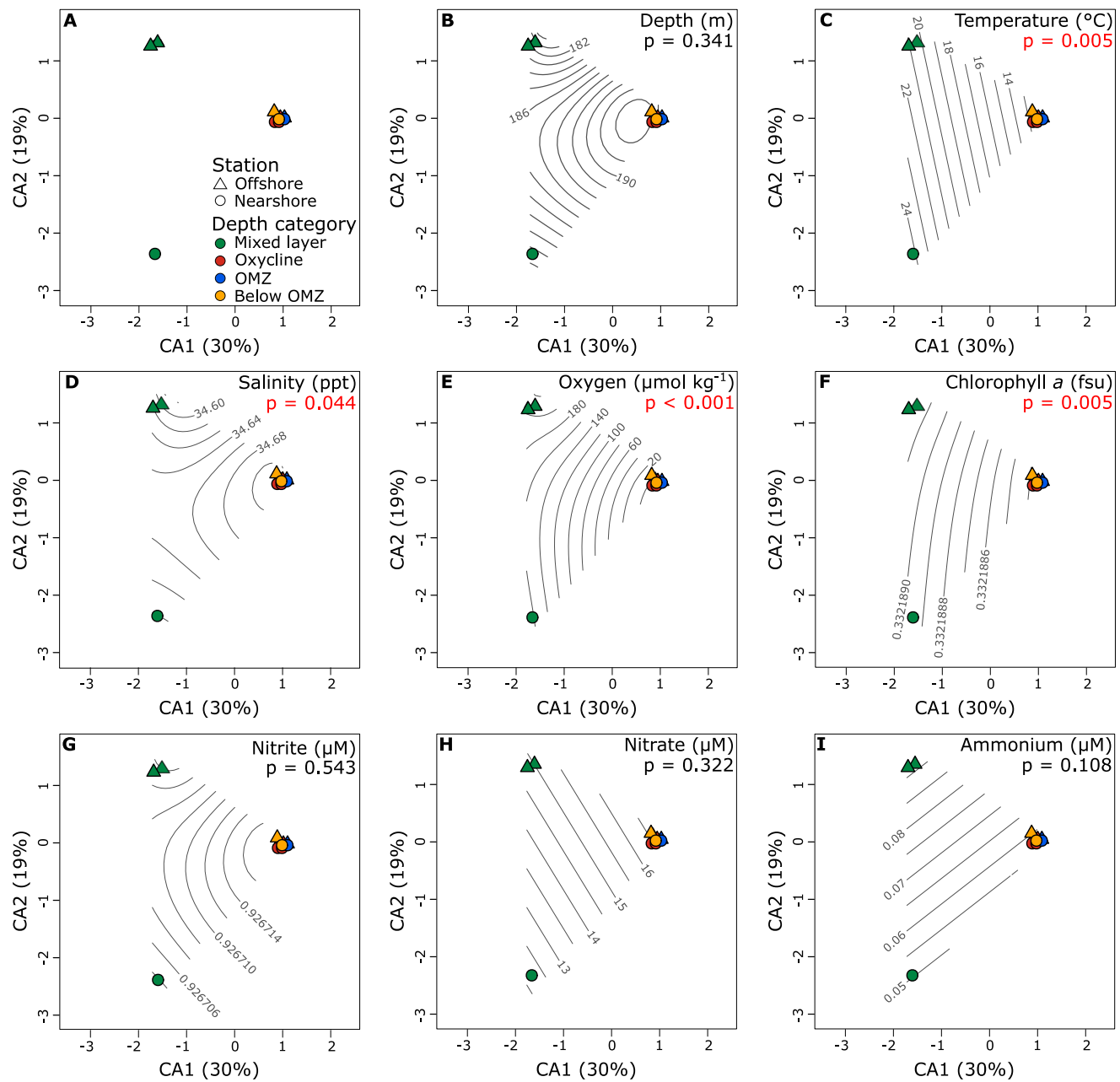

Supplement: Supplementary file 10 — Figure S9 [file 41396_2021_1143_MOESM10_ESM.pdf]
