# Supplementary material for: Viral community analysis in a marine oxygen minimum zone indicates increased potential for viral manipulation of microbial physiological state
Source: ISME J. 2021 Nov 6;16(4):972–82. doi: 10.1038/s41396-021-01143-1 (PMC8940887; doi:10.1038/s41396-021-01143-1)
Supplement: Supplementary file 6 — Figure S5 [file 41396_2021_1143_MOESM6_ESM.pdf]

Fig. S5

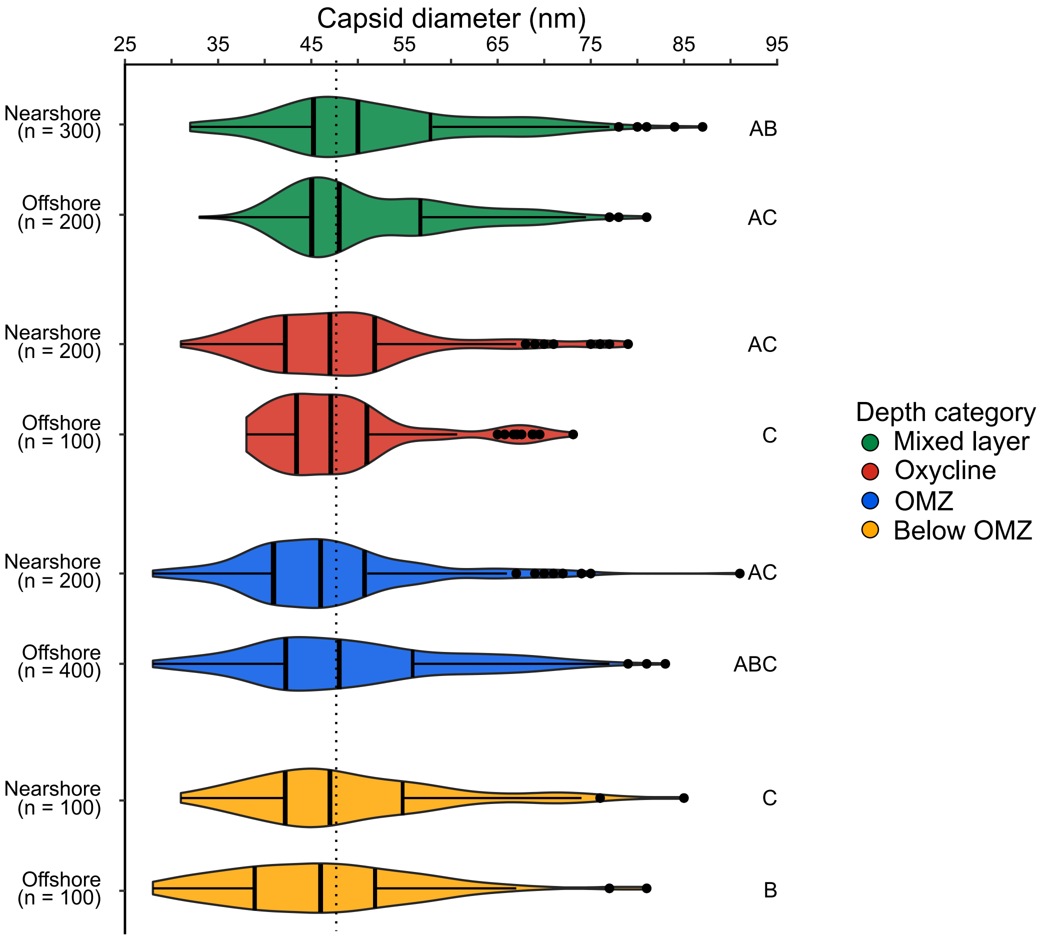

Figure S5. Violin plots comparing viral capsid diameter distributions in depth categories at each station. Left, middle, and right vertical lines in each plot correspond to the 25th, 50th (median), and 75th percentiles, respectively. Points represent outliers (> 2 standard deviations above the median for each sample). The dotted vertical line shows the global median capsid diameter for the entire dataset. Letters indicate significant differences between categories (ANOVA with Tukey's post-hoc test, $p < 0.001$ for all). The number of viruses included within each plot is given in parentheses.
