# Supplementary material for: Viral community analysis in a marine oxygen minimum zone indicates increased potential for viral manipulation of microbial physiological state
Source: ISME J. 2021 Nov 6;16(4):972–82. doi: 10.1038/s41396-021-01143-1 (PMC8940887; doi:10.1038/s41396-021-01143-1)
Supplement: Supplementary file 7 — Figure S6 [file 41396_2021_1143_MOESM7_ESM.pdf]

Fig. S6

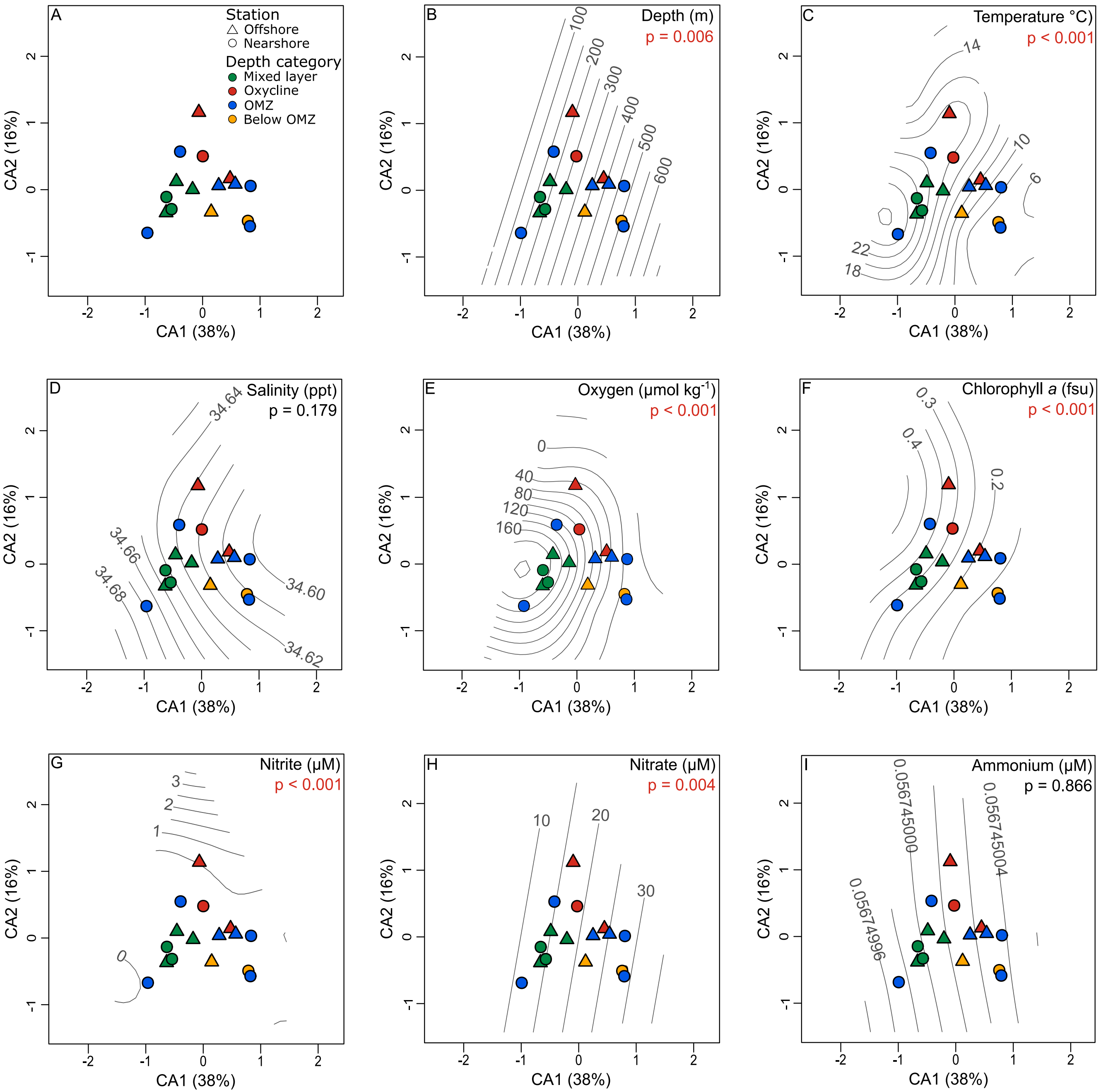

Figure S6. Correspondence analysis based on the distribution of viral capsid diameters with 5 nm bins for all samples (A). Response surfaces are shown for all environmental variables (B–I). P values highlighted in red are significant ($p < 0.05$). The percentage of inertia explained by CA1 and CA2 are reported on the axes.
