# Supplementary material for: Viral community analysis in a marine oxygen minimum zone indicates increased potential for viral manipulation of microbial physiological state
Source: ISME J. 2021 Nov 6;16(4):972–82. doi: 10.1038/s41396-021-01143-1 (PMC8940887; doi:10.1038/s41396-021-01143-1)
Supplement: Supplementary file 8 — Figure S7 [file 41396_2021_1143_MOESM8_ESM.pdf]

Fig. S7

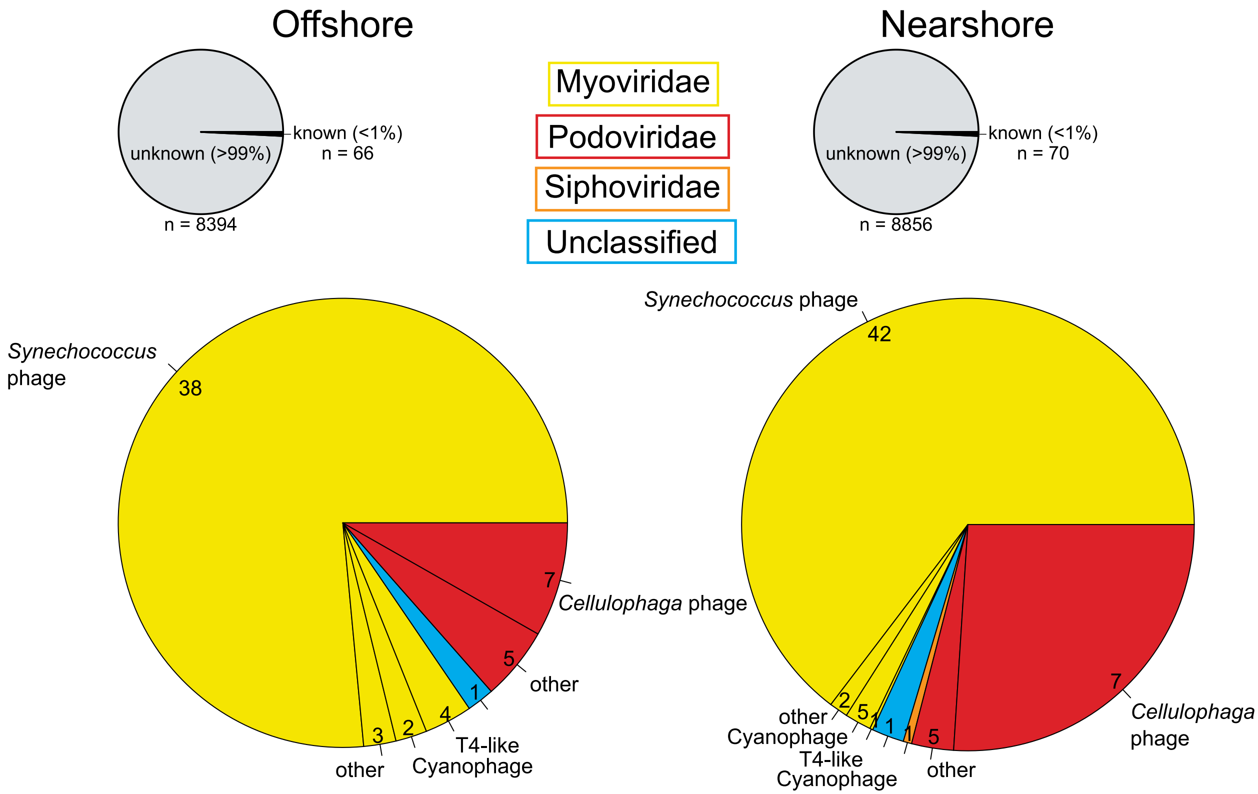

Figure S7. Taxonomic identification of all viral populations (smaller pie graph) and those that were taxonomically annotated (larger pie graph) for both stations. Those that made up <2% of each category are combined as "other". The area of each pie chart represents the relative abundance of each taxon, and the numbers inscribed in each section show the number of affiliated viral populations.
