# Supplementary material for: Viral community analysis in a marine oxygen minimum zone indicates increased potential for viral manipulation of microbial physiological state
Source: ISME J. 2021 Nov 6;16(4):972–82. doi: 10.1038/s41396-021-01143-1 (PMC8940887; doi:10.1038/s41396-021-01143-1)
Supplement: Supplementary file 9 — Figure S8 [file 41396_2021_1143_MOESM9_ESM.pdf]

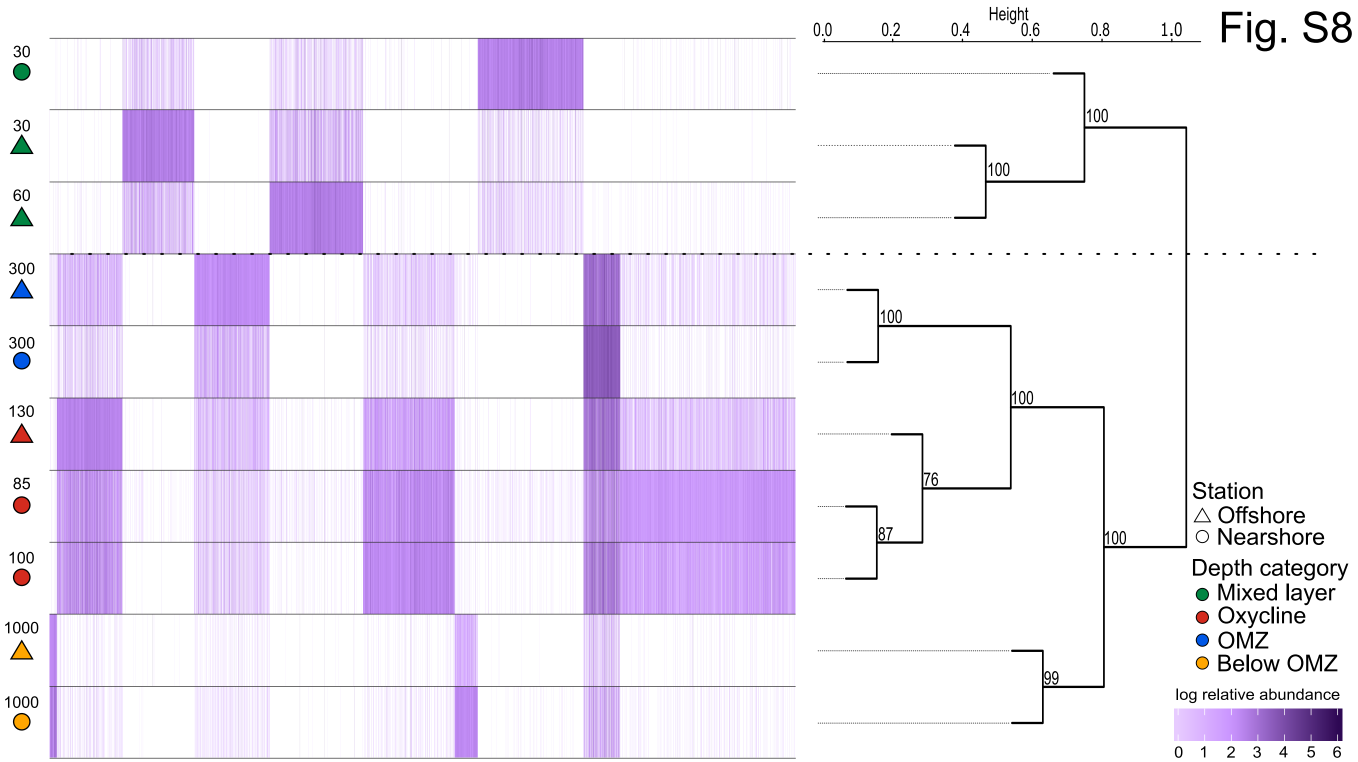

Figure S8. Heatmap based on normalized relative abundances of viral populations. Rows represent samples, labelled by station, depth category, and depth. Columns represent viral populations with the normalized relative abundance (log10 transformed) shown in purple. Hierarchical clustering of samples represented by the dendrogram revealed two clusters, denoted by the dotted line. Numbers on dendrogram nodes represent approximately unbiased bootstrapping values, based on 100 permutations.
