# Supplementary material for: Viral community analysis in a marine oxygen minimum zone indicates increased potential for viral manipulation of microbial physiological state
Source: ISME J. 2021 Nov 6;16(4):972–82. doi: 10.1038/s41396-021-01143-1 (PMC8940887; doi:10.1038/s41396-021-01143-1)
Supplement: Supplementary file 11 — Figure S9 [file 41396_2021_1143_MOESM11_ESM.pdf]

Fig. S9 (cont.)

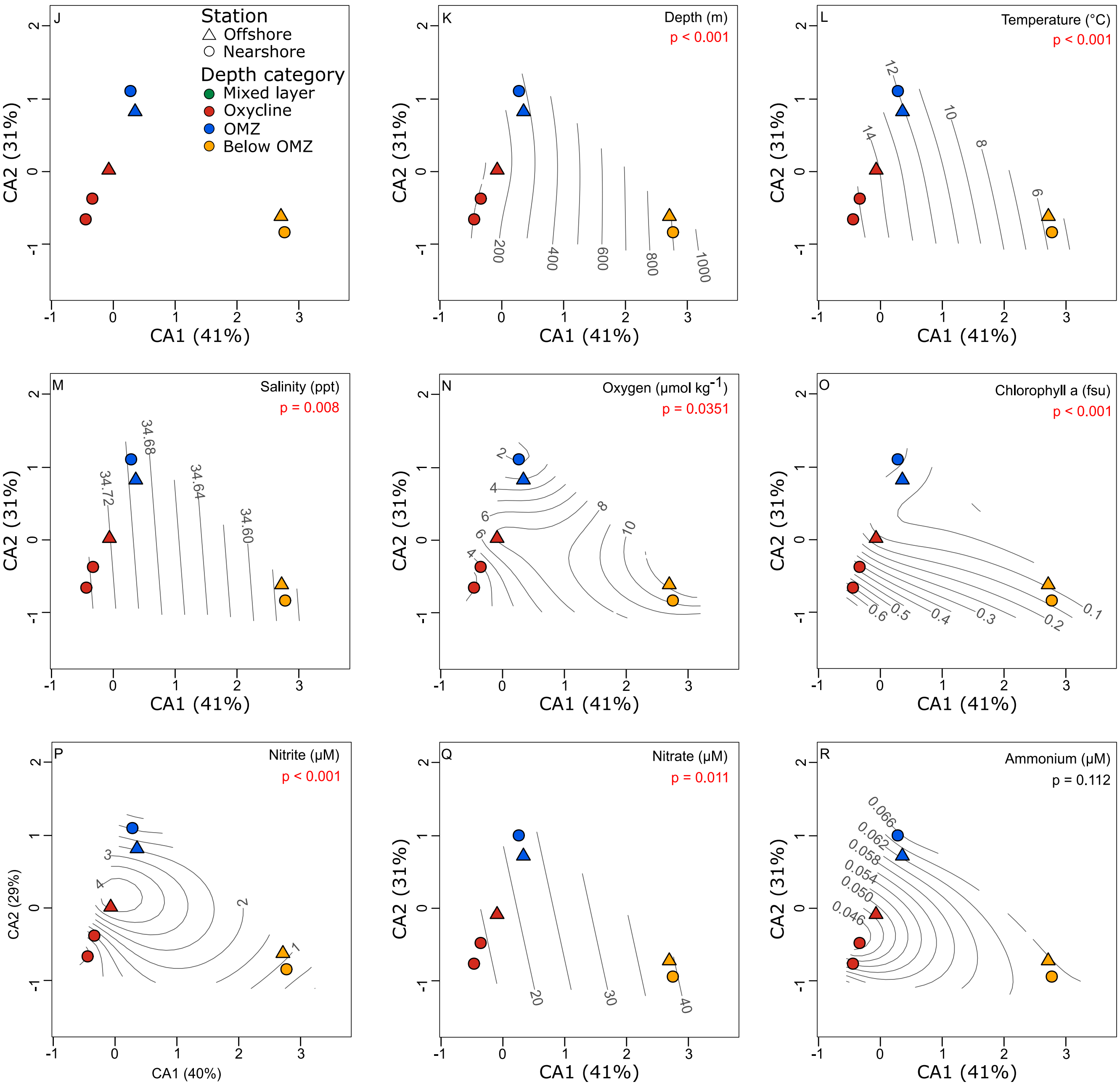

Figure S8. Correspondence analysis based on the relative abundance of viral populations for all samples (A) and the subset of low-oxygen samples (J). Response surfaces are shown for each environmental variable for all samples (B–I) and the sample subset (K–R). P values highlighted in red are significant ($p < 0.05$). The percentage of inertia explained by CA1 and CA2 are reported on the axes.
