# Supplementary material for: Viral community analysis in a marine oxygen minimum zone indicates increased potential for viral manipulation of microbial physiological state
Source: ISME J. 2021 Nov 6;16(4):972–82. doi: 10.1038/s41396-021-01143-1 (PMC8940887; doi:10.1038/s41396-021-01143-1)
Supplement: Supplementary file 12 — Figure S10 [file 41396_2021_1143_MOESM12_ESM.pdf]

Fig. S10

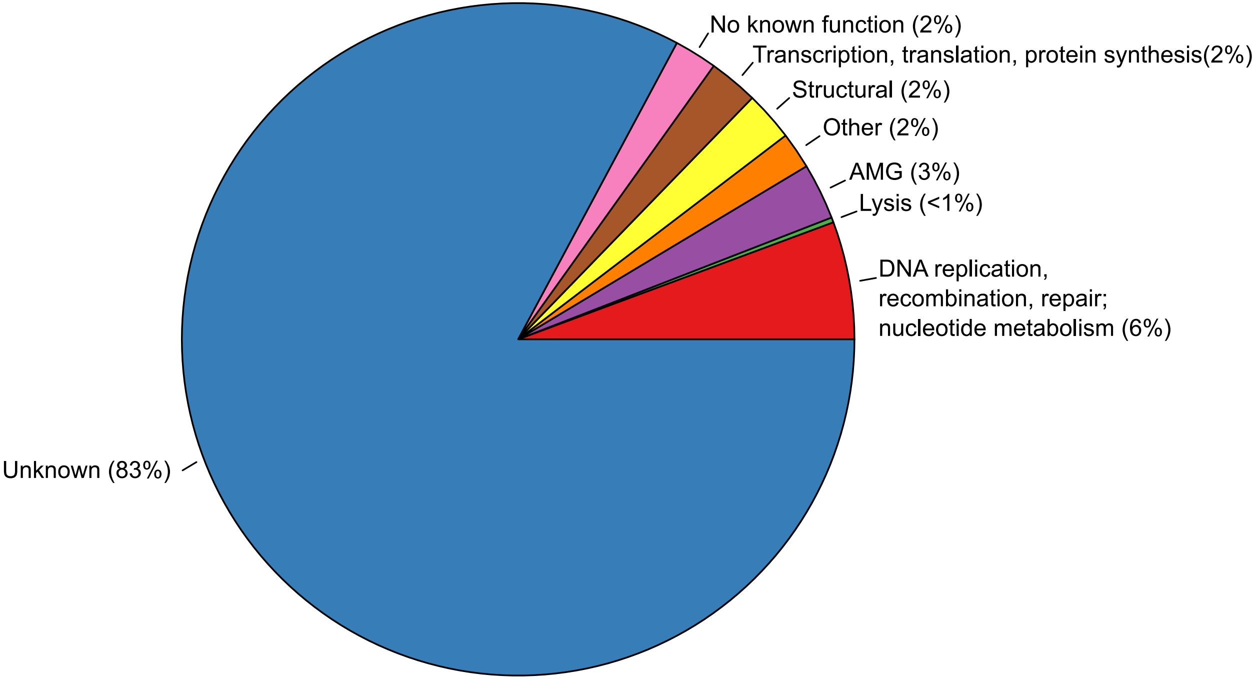

Figure S10. Relative abundance of all PFAM categories detected within viral populations in this study. “No known function” indicates predicted genes with hits to the KEGG database, but whose function has not been determined experimentally.
