# Supplementary material for: Viral community analysis in a marine oxygen minimum zone indicates increased potential for viral manipulation of microbial physiological state
Source: ISME J. 2021 Nov 6;16(4):972–82. doi: 10.1038/s41396-021-01143-1 (PMC8940887; doi:10.1038/s41396-021-01143-1)
Supplement: Supplementary file 13 — Figure S11 [file 41396_2021_1143_MOESM13_ESM.pdf]

Fig. S11

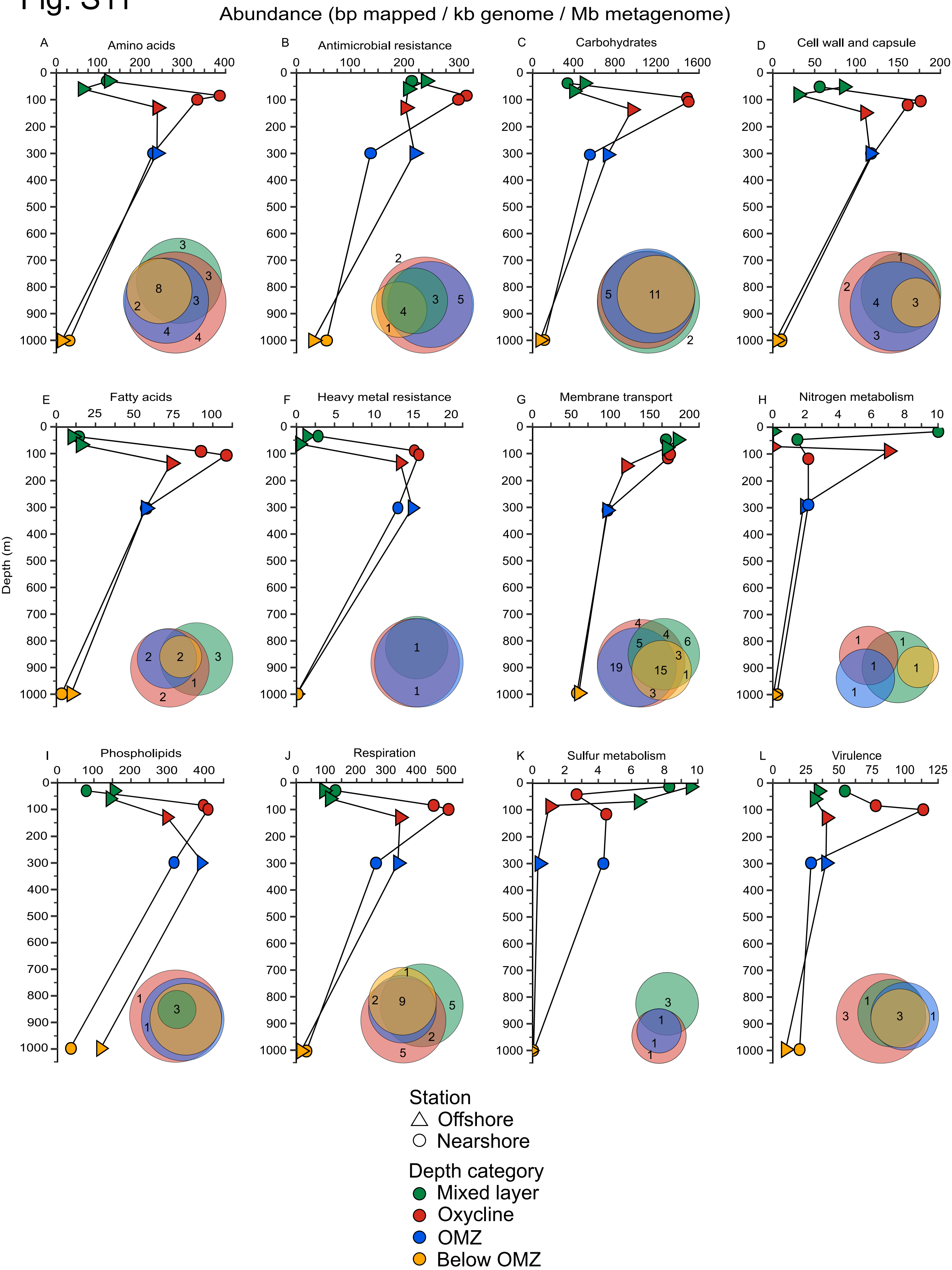

Figure S11. Depth profiles of additional AMG categories. Euler diagram insets represent the number of PFAMs unique and shared by depth category.
