# Supplementary material for: Viral community analysis in a marine oxygen minimum zone indicates increased potential for viral manipulation of microbial physiological state
Source: ISME J. 2021 Nov 6;16(4):972–82. doi: 10.1038/s41396-021-01143-1 (PMC8940887; doi:10.1038/s41396-021-01143-1)
Supplement: Supplementary file 14 — Figure S12 [file 41396_2021_1143_MOESM14_ESM.pdf]

Fig. S12

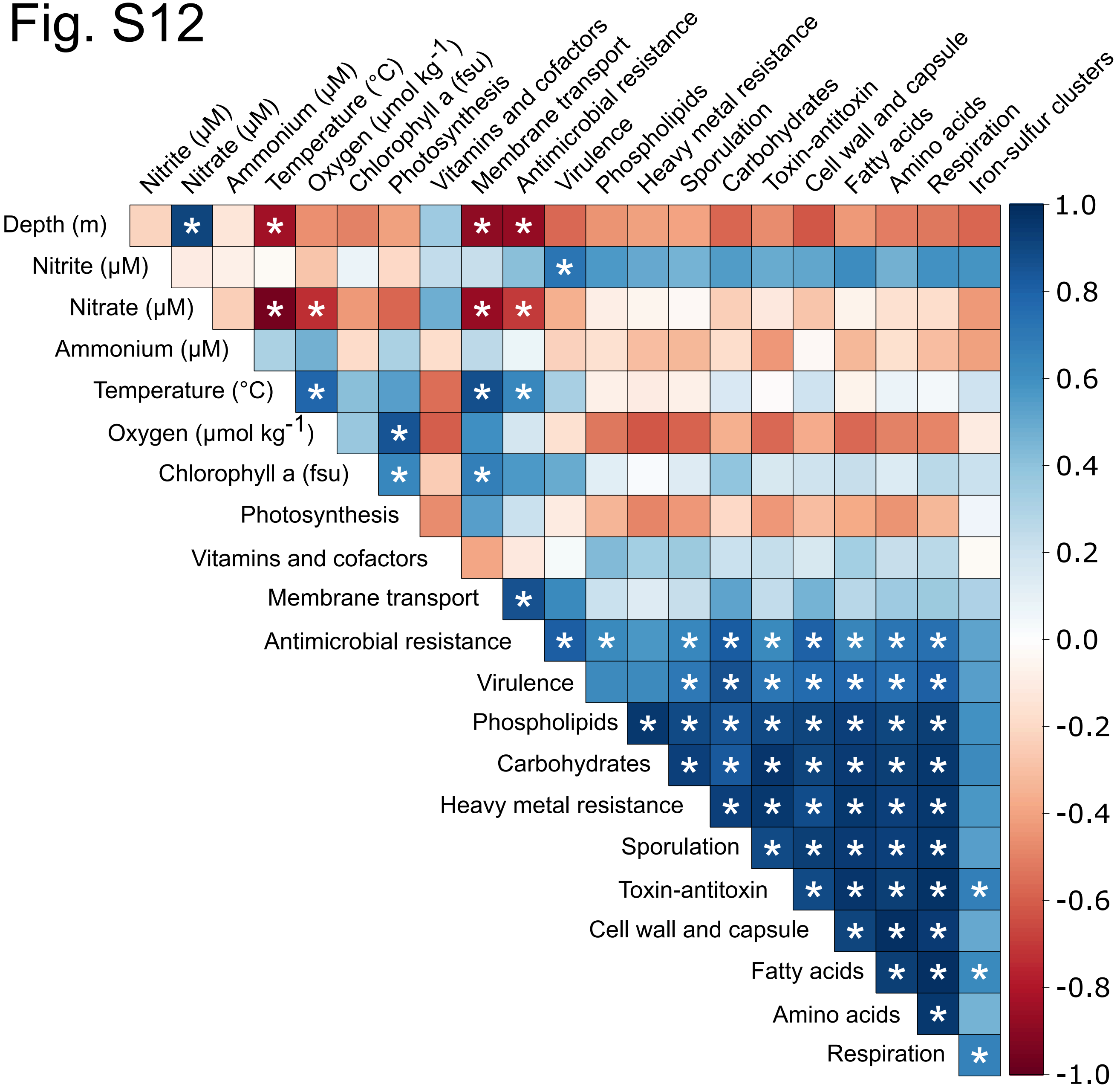

Figure S12. Matrix of Pearson correlations between environmental variables and AMG categories. Colors represent Pearson correlation coefficients. * indicate significant correlations ($p < 0.05$).
